# Solution of Radiative Transfer Equation with a Continuous and Stochastic Varying Refractive Index by Legendre Transform Method

**DOI:** 10.1155/2014/814929

**Published:** 2014-06-09

**Authors:** M. Gantri

**Affiliations:** Unit of Thermal Radiation, Department of Physics, Faculty of Sciences of Tunis, Tunis El-Manar University, 1060 Tunis, Tunisia

## Abstract

The present paper gives a new computational framework within which radiative transfer in a varying refractive index biological tissue can be studied. In our previous works, Legendre transform was used as an innovative view to handle the angular derivative terms in the case of uniform refractive index spherical medium. In biomedical optics, our analysis can be considered as a forward problem solution in a diffuse optical tomography imaging scheme. We consider a rectangular biological tissue-like domain with spatially varying refractive index submitted to a near infrared continuous light source. Interaction of radiation with the biological material into the medium is handled by a radiative transfer model. In the studied situation, the model displays two angular redistribution terms that are treated with Legendre integral transform. The model is used to study a possible detection of abnormalities in a general biological tissue. The effect of the embedded nonhomogeneous objects on the transmitted signal is studied. Particularly, detection of targets of localized heterogeneous inclusions within the tissue is discussed. Results show that models accounting for variation of refractive index can yield useful predictions about the target and the location of abnormal inclusions within the tissue.

## 1. Introduction

A special attention in diffuse optical tomography is focused on the development of methods for detection of photons providing the information concerning optical parameters of the explored medium. This gives the targets of localized nonhomogeneous inclusion arising in tissues due to various pathologies, like tumor formation, local increase in blood volume, and other abnormalities [1–4]. In radiative transfer theory, the most used parameters in modeling laser radiation interaction with biological tissue are absorption and scattering [5–7]. However some other studies evoked a significant variation of refractive index of abnormal biological tissues especially in the near infrared range. More precisely, experimental results [8, 9] showed that the tissue of malignant tumors could manifest an increase of the refractive index which can attain until 10% of that of a normal tissue which encircles them. So, medical imaging by diffuse optical tomography should take advantage from the emergence of a third contrast parameter which is the refractive index. This led to the appearance of a big number of numerical and fundamental works in the field of radiative transfer in a varying refractive index biological medium. While the conventional radiative transfer equation (RTE) has been widely used to study interaction of near infrared radiation with biological media, there exist a number of works dealing with a modified radiative transfer equation in spatially varying refractive index media [10, 11]. Some of these papers are interested in varying refractive index biological tissues [12–14]. In the present paper, our first concern is to contribute to the usability of the radiative transfer theory in a potential optical tomography setting in medical imaging. At this level, studying the effect of refractive index on the transmitted light through a biological rectangular layer should be crucial. This could improve detectability of heterogeneous objects in a typical tomography scheme. However, it is important to note that in a varying refractive index medium, the rays are not straight lines but curves. So even in a rectangular geometry, the varying index radiative transfer equation displays the classical form of the angular derivative terms commonly appearing when dealing with spherical and cylindrical geometries with uniform refractive index [15–17]. This finding gives rise to the use of Legendre transform as a manner for modeling these terms. Although this technique was used by Sghaier et al. [17] in a uniform refractive index spherical domain as an innovative view to handle these terms, it prevails as useful in this contemporary problem. This fact is our second concern in this paper. So, we present a computational RTE-based model suitable for basic diffuse optical tomography forward problem with spatially varying refractive index biological medium. We treat angular derivative terms by using the Legendre integral transform technique. We investigate cases concerning optical tomography applications. Results concerning the effect of the refractive index variation on the detected signal are shown.

## 2. Mathematical Model

In this work, the radiative transfer equation in a human biological tissue is described by using a stationary varying refractive index RTE [18, 19]

(1) Ω→·∇Ψ(r→,Ω→)+(μa(r→)+μs(r→))Ψ(r→,Ω→)  +1n∇n·∇Ω→Ψ(r→,Ω→)−2n(Ω→·∇n)Ψ(r→,Ω→) =μs(r)4π∫04πP(Ω→,Ω′→)Ψ(r,Ω′→)d(Ω′→)+S(r→,Ω→),

where

- $\Psi \left(\vec{r},\vec{\Omega }\right)$ is the directional energetic radiance at the spatial position vector $\vec{r}=\left(x,y,z\right)$,
- $n\left(\vec{r}\right)$ is the refractive index distribution,
- $\mu_{a}\left(\vec{r}\right)$ and $\mu_{s}\left(\vec{r}\right)$ are the absorption and scattering coefficients, respectively,
- *c* = *c*_vac_/*n*_*r*_ is the ratio of speed of light in a vacuum, *c*_vac_, and the refractive index *n*_*r*_,
- the source term $S(\vec{r},\vec{\Omega })$ is an injected radiance at the medium's boundary,
- the phase function $P(\vec{\Omega },\vec{\Omega^{\prime }})$ describes the probability that, during a scattering event, a photon with direction $\vec{\Omega^{\prime }}$ is scattered in the direction $\vec{\Omega }$.

Equation (1) takes into account the fact that the rays are not straight lines but curves. It involves terms that illustrate the expansion or the contraction of the cross section of the tube of light rays in the medium.

For a two-dimensional problem and in Cartesian coordinate system of the *x*-*y* plane, the terms due to the refractive index variation can be expressed as

(2) 1n∇n·∇Ω→Ψ−2n(Ω→·∇n)Ψ =−1n∂n∂x(sinφ∂Ψ∂φ+2cos⁡φΨ)  +1n∂n∂y(cos⁡φ∂Ψ∂φ−2sinφΨ),

where cos⁡*φ* and sin*φ* are the Cartesian coordinates of the unit direction vector in the *x*-*y* plane. In fact we assume that the radiance of out of plane directions is negligible. By using notations *ξ* = cos⁡*φ* and *η* = sin*φ*, (2) displays the classical form of the angular redistribution term commonly appearing when dealing with spherical and cylindrical geometries with uniform refractive index [20]

(3) 1n∇n·∇Ω→Ψ−2n(Ω→·∇n)Ψ  =12n2{∂n2∂x∂∂ξ[(1−ξ2)Ψ]+∂n2∂y∂∂η[(1−η2)Ψ]}.

The angular redistribution terms will be noted

(4) $$\begin{array}{c} D_{x}=\frac{\partial }{\partial \xi }\left[\left(1-\xi^{2}\right)\Psi \right],\\ D_{y}=\frac{\partial }{\partial \eta }\left[\left(1-\eta^{2}\right)\Psi \right] \end{array}$$

so (3) becomes

(5) $$\begin{array}{c} \frac{1}{n}\nabla n\cdot \nabla_{\vec{\Omega }}\Psi -\frac{2}{n}\left(\vec{\Omega }\cdot \nabla n\right)\Psi =\frac{1}{2n^{2}}\left\{\frac{\partial n^{2}}{\partial x}D_{x}+\frac{\partial n^{2}}{\partial y}D_{y}\right\}. \end{array}$$

## 3. Numerical Method

In our numerical implementation, we use a rectangular domain which is divided into a set of *I* × *J* elementary uniform volumes Δ*V* = Δ*x*Δ*y*Δ*z* with a uniform unitary depth (Δ*z* = 1). The angular discretization is obtained through a discrete ordinate technique. This yields a set of *M* discrete directions, *φ*_*m*_, *m* = 1 ⋯ *M* giving a set of angular discrete direction cosines (*ξ*_*m*_, *η*_*m*_), *m* = 1 ⋯ *M*. An orientation depending on the incident ray direction is adopted for each cell [7]. Calculations are done by using integration of (1) over an elementary volume Δ*V* for each discrete direction. This gives

(6) Δyξm(Ψm,E−Ψm,W)+Δxηm(Ψm,N−Ψm,S)  +ΔxΔy(μa+μs)Ψm,P+1n(∂n∂xDm,x+∂n∂yDm,y) =ΔxΔy(Sm,P+μs∑m′=1,m′≠mMwm′pmm′Ψm′,P),

where *D*_*m*,*x*_ and *D*_*m*,*y*_ are the discrete angular derivative terms at the angular ordinates *ξ*_*m*_ and *η*_*m*_, respectively, and *w*_*m*_ is a weighting factor. The discrete term of Henyey-Greenstein phase function is written as

(7) $$\begin{array}{c} p_{mm^{\prime }}=\frac{1-g^{2}}{4\pi {\left(1+g^{2}-2g\left(\xi_{m}\xi_{m^{\prime }}+\eta_{m}\eta_{m^{\prime }}\right)\right)}^{3/2}}, \end{array}$$

where *g* is the anisotropy factor. If the direction cosines are positive, the directional radiance is known on the faces *W* and *S* and they are unknown on the faces *E* and *N* of the (*i*, *j*)-cell and also in the centre *P*. Therefore, we need two complementary relations to eliminate Ψ_*m*,*E*_ and Ψ_*m*,*N*_; this can be obtained by using interpolation formula

(8) $$\begin{array}{c} \Psi_{m,P}=\alpha \Psi_{m,E}+\left(1-\alpha \right)\Psi_{m,W},\\ \Psi_{m,P}=\alpha \Psi_{m,N}+\left(1-\alpha \right)\Psi_{m,S}, \end{array}$$

where *α* is an interpolation parameter. Using these relations, (6) becomes

(9) Δyξmα(Ψm,P−Ψm,W)+Δxηmα(Ψm,P−Ψm,S)  +ΔxΔy(μa+μs)Ψm,P+12n2[∂n2∂xDm,x+∂n2∂yDm,y] =ΔxΔy(Sm,P+μs∑m′=1,m′≠mM‍wm′pmm′Ψm′,P).

Theoretically, if we know the solution in the (*i*, *j*)-cell, we can do calculus over the cells (*i* + 1, *j*) and (*i*, *j* + 1) using the boundary conditions and the following relations:

(10) $$\begin{array}{c} \Psi_{m,W}\left(i+1,j\right)=\Psi_{m,E}\left(i,j\right);\;i=1\cdots I-1,\\ \Psi_{m,E}\left(i,j+1\right)=\Psi_{m,N}\left(i,j\right);\;j=1\cdots J-1. \end{array}$$

If the direction cosines are both positive, we get the following equation:

(11) Δyξmα(Ψm,i,j−Ψm,i−1,j)+Δxηmα(Ψm,i,j−Ψm,i,j−1)  +ΔxΔy(μa+μs)Ψm,i,j+12n2[∂n2∂xDm,x+∂n2∂yDm,y] =ΔxΔy(Sm,i,j+μs∑m′=1Mwm′pmm′Ψm′,i,j);

this gives

(12) Ψm,i,j=[Ψm,i,j−ni,j2cΔtΨm,i,j+ΔyξmαΨm,i−1,j+ΔxηmαΨm,i,j−1  +12ni,j2[Δy(ni,j2−ni−1,j2)Dm,x,i,j+Δy(ni,j2−ni,j−12)Dm,x,i,j]  +ΔxΔy(Sm,i,j+μs,i,j∑m′=1Mwm′pmm′Ψm′,i,j)] ×[Δyξmα+Δxηmα+ΔxΔy(μa,i,j+μs,i,j)]−1.

### 3.1. Numerical Treatment of Angular Derivative Terms with Finite Legendre Transform

As is explained in [17], we consider the following Legendre transforms:

(13) $$\begin{array}{c} L\left(\frac{\partial }{\partial \xi }\left[\left(1-\xi^{2}\right)\Psi \right],s\right)=\overset{1}{\underset{-1}{\int }}‍\frac{\partial }{\partial \xi }\left[\left(1-\xi^{2}\right)\Psi \right]P_{s}\left(\xi \right)d\xi ,\\ L\left(\frac{\partial }{\partial \eta }\left[\left(1-\eta^{2}\right)\Psi \right],s\right)=\overset{1}{\underset{-1}{\int }}‍\frac{\partial }{\partial \eta }\left[\left(1-\eta^{2}\right)\Psi \right]P_{s}\left(\eta \right)d\eta , \end{array}$$

where *P*_*m*_ is the *m* degree Legendre polynomial. According to Sturn-Liouville equation defining Legendre Polynomials, we can write

(14) ∫−11∂∂η[(1−ξ2)ψ]Ps(ξ)dξ  =s(s+1)2s+1[∫−11ΨPs+1(ξ)dξ−∫−11ΨPs−1(ξ)dξ],∫−11‍∂∂η[(1−ξ2)ψ]Ps(η)dη  =s(s+1)2s+1[∫−11‍ΨPs+1(η)dη−∫−11‍ΨPs−1(η)dη].

To obtain derivative terms, we make use of numerical quadrature:

(15) ∑m=1MwmDξ,mPs(ξm)  =s(s+1)2s+1[∑m=1M‍wmPs+1(ξm)−∑m=1M‍wmPs−1(ξm)],s=1,…,M−1,∑m=1M‍wmDη,mPs(ηm)  =s(s+1)2s+1[∑m=1M‍wmPs+1(ηm)−∑m=1M‍wmPs−1(ηm)],s=1,…,M−1.

The above systems of equations are closed by the obvious relations:

(16) $$\begin{array}{c} \overset{M}{\underset{m=1}{\sum }}‍w_{m}D_{\xi ,m}=\overset{1}{\underset{-1}{\int }}\frac{\partial }{\partial \eta }\left[\left(1-\xi^{2}\right)\psi \right]d\left(\xi \right),\\ \overset{M}{\underset{m=1}{\sum }}‍w_{m}D_{\eta ,m}=\overset{1}{\underset{-1}{\int }}\frac{\partial }{\partial \eta }\left[\left(1-\eta^{2}\right)\psi \right]d\left(\eta \right). \end{array}$$

So discrete derivative terms in the (*i*, *j*)-cell into the medium can be obtained by solving the linear algebraic equations

(17) $$\begin{array}{c} A_{\xi }{\hat{D}}_{\xi ,i,j}=B_{\xi ,i,j},\;\;A_{\eta }{\hat{D}}_{\eta ,i,j}=B_{\eta ,i,j}, \end{array}$$

where

(18) Aξ=(w1w2…wMw1P1(ξ1)w2P1(ξ2)…wMP1(ξM)⋮⋮⋮⋮w1PM−1(ξ1)w2PM−1(ξ2)…wMPM−1(ξM)),D^η,i,j=(Dξ,1,i,jDξ,2,i,j⋮Dξ,M,i,j),Bη,i,j=(023∑m′=1M‍wm′ψm′,i,j(P2(ξm′))−(P0(ξm′))⋮M(M−1)2M−1∑m′=1M‍wm′ψm′,i,j(PM(ξm′)−PM−2(ξm′)))

and $A_{\eta },{\hat{D}}_{\eta ,i,j},B_{\eta ,i,j}$ are straightforward by replacing *ξ* by *η*.

A set of weights are judiciously chosen with an equally spaced distribution of angular ordinates (*ξ*, *η*). The matrices *A*_*ξ*_ and *A*_*η*_ must be regular and they are inversed each only once in all the calculation process.

To solve (12), we use successive iterations to actualise the implicit internal source term in the right member. So, this gives

(19) Ψm,i,jk+1=[ΔyξmαΨm,i−1,jk+1+ΔxηmαΨm,i,j−1k+1  +12ni,j2[Δy(ni,j2−ni−1,j2)Dm,x,i,j+Δy(ni,j2−ni,j−12)Dm,x,i,j]  +ΔxΔy(Sm,i,jk+1+μs,i,j∑m′=1Mwm′pmm′Ψm′,i,jk)] ×[Δyξmα+Δxηmα+ΔxΔy(μa,i,j+μs,i,j)]−1.

The iteration process is repeated until a convergence criterion is attempted. To improve convergence speed, we use a successive overrelaxation method. So the updated value Ψ_*m*,*i*,*j*_^*k*+1^ is a linear combination of the iterated value Ψ_*m*,*i*,*j*_^*k*^ and the previously computed value

(20) $$\begin{array}{c} {\left(\Psi_{m,i,j}^{k+1}\right)}_{\text{updated}}=\rho \Psi_{m,i,j}^{k}+\left(1-\rho \right)\Psi_{m,i,j}^{k+1}, \end{array}$$

where *ρ* is a relaxation parameter whose value is usually between 1 and 2. The solution is obtained when the relative discrepancy value

(21) $$\begin{array}{c} \varepsilon =\frac{\Psi_{m,i,j}^{k+1}-\Psi_{m,i,j}^{k}}{\Psi_{m,i,j}^{k}} \end{array}$$

is smaller than a tolerance value. In all our calculus, we have taken 10^−8^ as a tolerance value. As initial condition, we take a field of null radiance. Also, all our calculations are done in the case of interpolation diamond scheme (*α* = 0.5).

If the direction cosines are not both positive, the precedent equations are valid provided that the orientation WESN of cells is done according to the direction of propagation [7]. In all our investigations, the injected power source is assumed to be equivalent to a forward collimated monochromatic intensity placed at a source point on the middle of the bottom side of the boundary. Results shown below are obtained by using a continuous wave source with a uniform equivalent intensity value of 50 mW·cm^−1^.

### 3.2. Boundary Conditions

On the boundary, the radiance is the sum of the external source contribution and the partly reflected radiance due to the refractive index mismatch at the boundary

- $\Psi \left(\vec{r},\vec{\Omega }\right)=S\left(\vec{r_{b}},\vec{\Omega }\right)+R\cdot \Psi (\vec{r},{\vec{\Omega }}_{\text{ref}})$;
- ${\vec{u}}_{b}\cdot \vec{\Omega }<0$ and ${\vec{u}}_{b}\cdot {\vec{\Omega }}_{\text{ref}}=-{\vec{u}}_{b}\cdot \vec{\Omega }$,

where $\vec{r_{b}}$ is a position on the boundary and ${\vec{u}}_{b}$ is an outer normal unit vector. The reflectivity *R* can be calculated for each direction using Fresnel's relations.

To present our results, we use the detected fluence rate which is given in a (*i*_*d*_, *j*_*d*_)-detector point on the boundary as

(22) $$\begin{array}{c} \Phi_{d}=\overset{M}{\underset{m=1}{\sum }}‍\left(1-R_{m,i_{d},j_{d}}\right)w_{m}\Psi_{m,i_{d},j_{d}}, \end{array}$$

with

(23) Rm,id,jd={1 if  φm>arcsin(nairnid,jd)12[(nid,jdcos⁡φm−naircos⁡φtnid,jdcos⁡φm+naircos⁡φt)2   +(nid,jdcos⁡φt−naircos⁡φmnid,jdcos⁡φt+naircos⁡φm)2], else,φt=arcsin(nairsinφmnid,jd), nair≈1.

Also, we make use of a normalized detected fluence rate defined as

(24) $$\begin{array}{c} \Phi_{N}=\frac{\Phi_{d}}{\left(1/D\right)\sum_{d=1}^{D}w_{d}^{\prime }\Phi_{d}}, \end{array}$$

where *D* is the number of the detector points on one side of the boundary and *w*_*d*_′ is a weighting factor from the generalized trapezoidal integration rule.

In all calculations, we have used 28 detector points on each side. Also, all calculus is carried out by using 16 uniformly distributed discrete directions and a space grid of 121 × 121 cells.

## 4. Results and Discussion

### 4.1. Continuous Varying Refractive Index Medium

**Figure 1:**
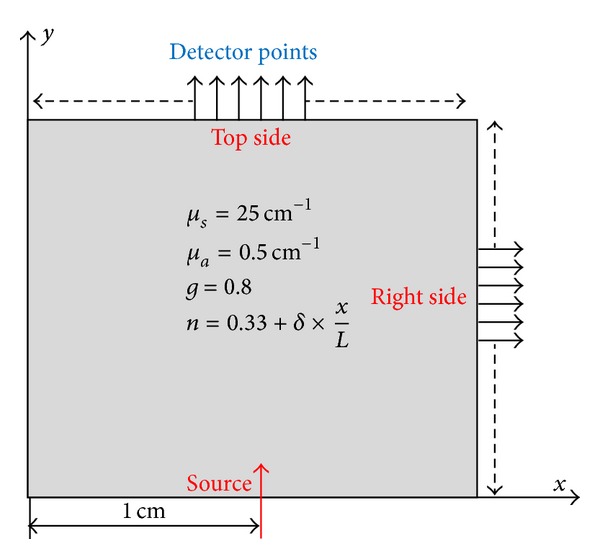
A test-medium with background properties and detector points.

In this investigation, we study near infrared radiation transport in a rectangular medium exposed to a continuous collimated source which is placed on the bottom side of the boundary.  Figure 1 shows the considered medium; it is assumed to be 2 × 2 cm sized with varying refractive index. Within the medium, we consider a *x*-axis linear refractive index variation with different gradient values. To show the effect of the refractive index on detected fluence rate, we have used a weakly absorbing and forward scattering background medium whose optical parameters are shown in Figure 1.

**Figure 2:**
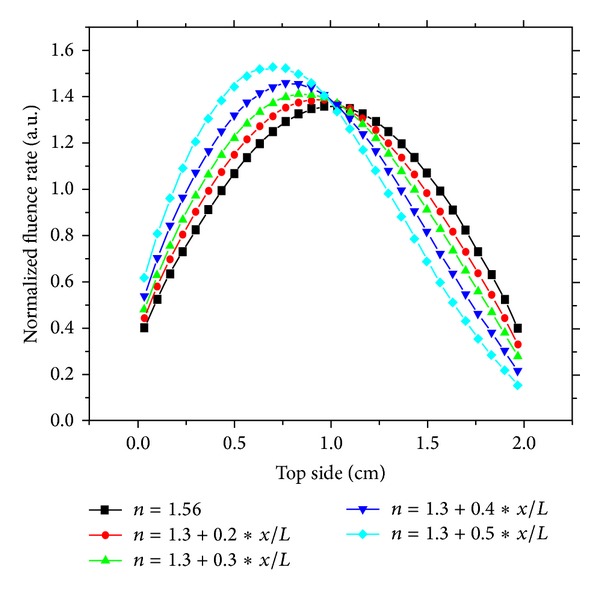
Response of a continuous varying refractive index medium detected fluence rate on the top side: gradient effect.

**Figure 3:**
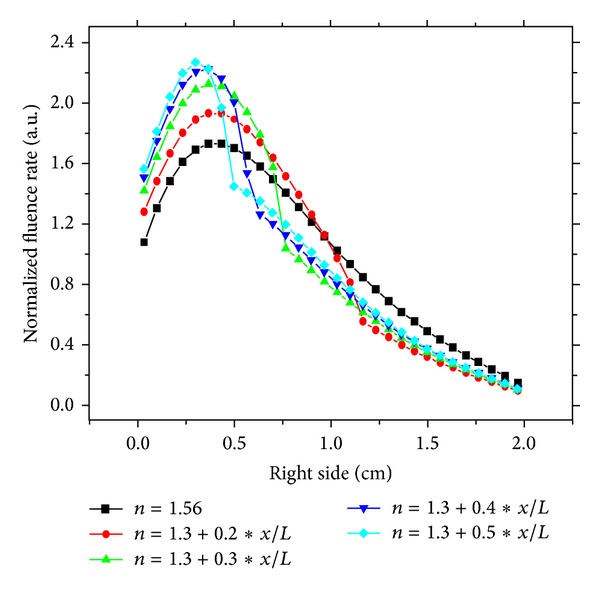
Response of a continuous varying refractive index medium detected fluence rate on the right side: gradient effect.

Figures 2 and 3 represent the response of the medium through the detected signal on the top and right side of the boundary in the case of linear variation of refractive index. We note that the response of the medium varies linearly according to the gradient of refractive index. The maximum transmission moves to regions of height index (Figure 2). Indeed, according to boundary conditions of the medium (Descartes Laws) the transmission window increases proportionally of refractive index between the medium and outside medium (air).

On the other hand, the right side (Figure 3) shows the response of the medium detected on the right side of the boundary in the case of linear and parabolic variation, respectively. The detected fluence rate curves presents distinguished effect of refractive index gradient in linear case. The transmission zone on the boundary increases with decreasing gradient values. Even though most detected transmission is obtained near the source, a weak gradient can augment transmitted radiation relatively far from the source while high values of gradient can block transmitted radiation within the medium.

### 4.2. Effect of Stochastic Varying Refractive Index

**Figure 4:**
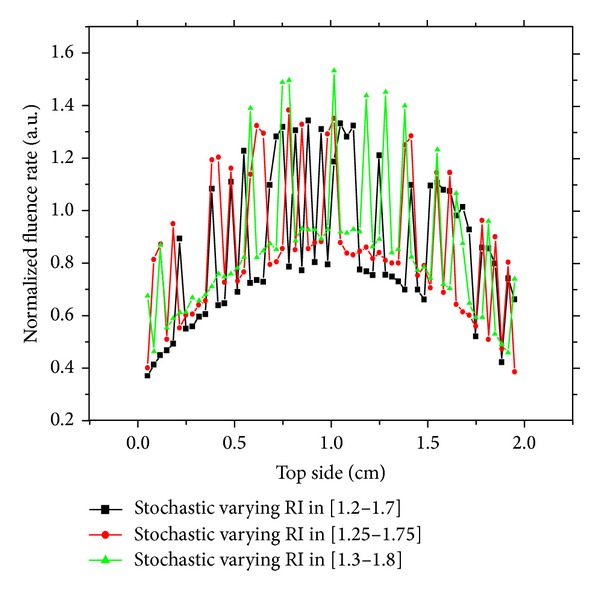
Response of stochastic varying refractive index medium on the top side.

**Figure 5:**
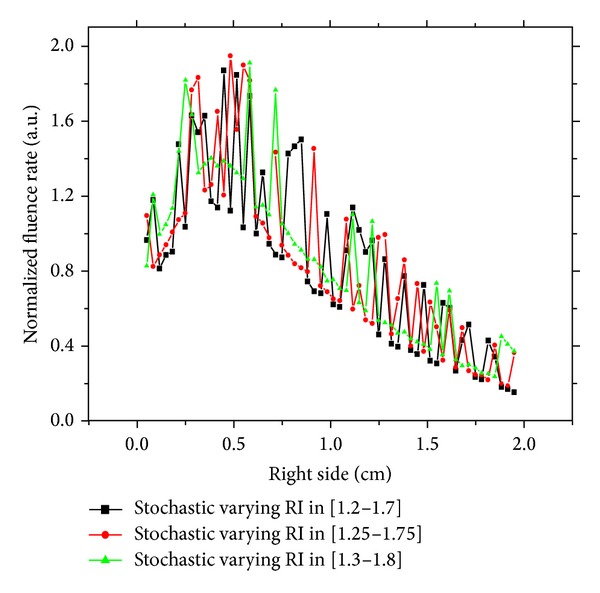
Response of stochastic varying refractive index medium on the right side.

In this investigation, we keep the same medium precedent, but we will study the stochastic variation refractive index. We control this variation by uniform random fixing of a given interval. The other optical properties are the same as in the precedent investigation. In such cases, there is an obvious effect of the heterogeneity on the detected signal especially when the refractive index is increased or decreased by 10% in a stochastic way. The detected signal on different sides of the medium is shown in Figures 4 and 5. There is a significant effect of this variation on the detected signal on the top side and right side, because there is strong perturbation in medium. These findings highlight the potential of refractive index as a possible detection parameter of a tumor in a surrounding safe tissue. These findings open prospects for further study of the effect of local refractive variation in domain and frequency-domain schemes in diffuse optical tomography.

## 5. Conclusion

This study attempted to develop a computational way helping in detection of abnormalities in a biological tissue. This should enable predictions of eventual tumor existence when using a diffuse optical tomography scheme. The used model is based on stationary radiative transfer equation including a possible continuous and stochastic variation of refractive index. In particular, computational technique of Legendre transform is extended to handle angular derivative terms arising by the varying refractive index consideration. The obtained computational model is implemented to investigate some practical situations in diffuse optical tomography (DOT) setting. Obtained results showed that variation of refractive index can yield useful predictions about the target and the location of abnormal inclusions within the tissue. These findings open prospects for further study of the effect of local refractive variation in time-domain and frequency-domain schemes in diffuse optical tomography.
